# Focal Adhesion Kinase (FAK) Over-Expression and Prognostic Implication in Pediatric Hepatocellular Carcinoma

**DOI:** 10.3390/ijms21165795

**Published:** 2020-08-12

**Authors:** Paola Francalanci, Isabella Giovannoni, Cristiano De Stefanis, Ilaria Romito, Chiara Grimaldi, Aurora Castellano, Valentina D’Oria, Rita Alaggio, Anna Alisi

**Affiliations:** 1 Pathology Unit, Bambino Gesù Children’s Hospital and IRCCS, 00165 Rome, Italy; paola.francalanci@opbg.net (P.F.); isabella.giovannoni@opbg.net (I.G.); rita.alaggio@opbg.net (R.A.); 2 Core Facilities, Bambino Gesù Children’s Hospital and IRCCS, 00165 Rome, Italy; cristiano.destefanis@opbg.net; 3 Research Unit of Molecular Genetics of Complex Phenotypes, Bambino Gesù Children’s Hospital and IRCCS, 00165 Rome, Italy; ilaria.romito@opbg.net; 4 Department of Pediatric Surgery and Transplantation, Bambino Gesù Children’s Hospital and IRCCS, 00165 Rome, Italy; chiara.grimaldi@opbg.net; 5 Department of Pediatric Hematology and Oncology, Bambino Gesù Children’s Hospital and IRCCS, 00165 Rome, Italy; aurora.castellano@opbg.net; 6 Microscopy Unit, Bambino Gesù Children’s Hospital and IRCCS, 00165 Rome, Italy; valentina.doria@opbg.net

**Keywords:** pediatric hepatocellular carcinoma, focal adhesion kinase, enhancer of Zeste homolog 2, β-Catenin, cirrhosis, normal liver

## Abstract

Focal adhesion kinase (FAK) is over-expressed and is correlated with aggressiveness in adult hepatocellular carcinoma (HCC). Inhibition of FAK decreases HCC invasiveness by down-regulating Enhancer of Zeste homolog 2 (EZH2), an epigenetic controller, that acts in transcriptional repression of a large number of genes via histone 3 methylation of lysine 27 (H3K27me3). Here, we investigated the hepatic expression of total FAK, EZH2, H3K27me3, and proliferating cell nuclear antigen (PCNA) in 17 pediatric HCCs and 8 healthy livers (CTRL). Quantitative imaging analysis showed that FAK gene/protein expression is up-regulated in HCCs compared to CTRL and, among tumor samples the levels of this gene/protein are significantly higher in cirrhotic HCCs than in a healthy milieu. Accordingly, the protein levels of EZH2 were also significantly increased in HCCs from a cirrhotic background. Intriguingly, the protein expression of FAK, EZH2, and PCNA significantly inversely correlated with tumor size. Finally, in HCC samples, mainly in cirrhotic background, the up-regulation of FAK gene positively correlated with that observed in β-Catenin gene. Conclusion: FAK gene/protein is over-expressed in pediatric HCCs concomitantly to EZH2 protein and β-Catenin gene, with a more significant up-regulation in a cirrhotic background. This triad of interactors deserves further investigations for translational application.

## 1. Introduction

The primary pediatric liver malignancies comprise 1% to 2% of all childhood tumors. Hepatocellular carcinoma (HCC) is the second most common primary liver malignancy of childhood (27%) [1]. Pediatric HCC may arise in the context of cirrhosis related to underlying metabolic or genetic diseases or in livers without chronic disease (CLD) [2,3].

Pediatric HCC independent from its clinical background is considered a highly aggressive neoplasm and liver transplant may represent the only therapeutic option in case of unresectable tumors. Little information is available on cytogenetic background of pediatric HCC. Several molecular aberrations that characterized adult HCC have been found also in pediatric HCC, while for other pathways, such as angiogenesis signaling, there are no data in children [2]. This suggests that some pediatric HCCs could be treated as their adult counterpart. In particular, as in adult HCC, a subset of pediatric HCC is characterized by alterations in WNT signalling, β-Catenin (CTNNB1), and telomerase reverse transcriptase (TERT) genes [4]. Profiling expression of β-Catenin protein (β-Cat), its downstream target glutamine synthetase (GS), and its interactor Glypican-3 (GPC3) could be helpful in the identification of altered Wnt/β-Cat pathways. Thus, immunohistochemical co-expression of β-Cat, GS, and GPC3 observed in HCC suggests that GPC3-induced activation of the Wnt/β-Cat pathway is responsible for the development of malignant hepatocellular tumors [5].

In the cirrhotic context, the development of HCC implies both fibrogenetic mechanism and interaction between necro-inflammatory and regenerative events. Nevertheless, only about 30% of pediatric cases of HCC are associated with cirrhosis or pre-existing liver abnormality [6]. Therefore, it is reasonable to hypothesize that other genetic and epigenetic changes may be involved in pathogenesis of HCC in healthy liver in the pediatric age [4].

Among others, the Enhancer of Zeste homolog 2 (EZH2), an epigenetic controller that acts in transcriptional repression of a large number of genes via histone 3 methylation of lysine 27 (H3K27me3), is up-regulated in HCC [7]. Increased expression of EZH2 is reported to be concomitant with increased expression of H3K27me3 suggesting that EZH2 might catalyse trimethylation of H3K27 in HCCs [8]. High levels of both EZH2 and H3K27me3 are associated to a more aggressive HCCs with morphologic markers of poor prognosis. Cai et al., [9] also reported that EZH2 could serve as a promising immune-marker of HCCs in the diagnostic work-up of liver lesions. Moreover, we found that the expression/activity of EZH2 was regulated both in vitro and in vivo models of HCC by a non-receptor tyrosine kinase called focal adhesion kinase (FAK) [10]. FAK protein expression was strongly correlated to aggressive behavior and poor prognosis in HCC [11,12]. Interestingly, Kan et al. [13] reported a 26.1% of HCCs harboring amplification of the gene encoding for FAK.

The relationship between FAK and EZH2 has been well defined in a few tumors. Indeed, a previous study demonstrated that FAK activity is influenced by EZH2 in dendritic cells [14]. Moreover, Zhou et al. [15] reported a correlated over-expression of methyltransferase EZH2, FAK and 397 tyrosine-phosphorylated form of FAK in endometrial carcinomas, which was associated with high histologic grade, angiolymphatic invasion, lymph node metastasis, myometrial invasion, and cervical involvement, thus predicting a more aggressive behavior. However, neither the expression of FAK nor that of EZH2 and their relevance as tissue biomarkers have been investigated before in pediatric HCCs.

In the present study, we investigate the expression and activity of FAK, and its correlation with the expression of EZH2, H3K27me3, and proliferating cell nuclear antigen (PCNA), and its possible prognostic value, in 17 pediatric HCCs and 8 age-matched healthy livers.

## 2. Results

### 2.1. Total Levels of FAK Protein/Gene and Activity Increase in Pediatric HCCs

**Figure 1:**
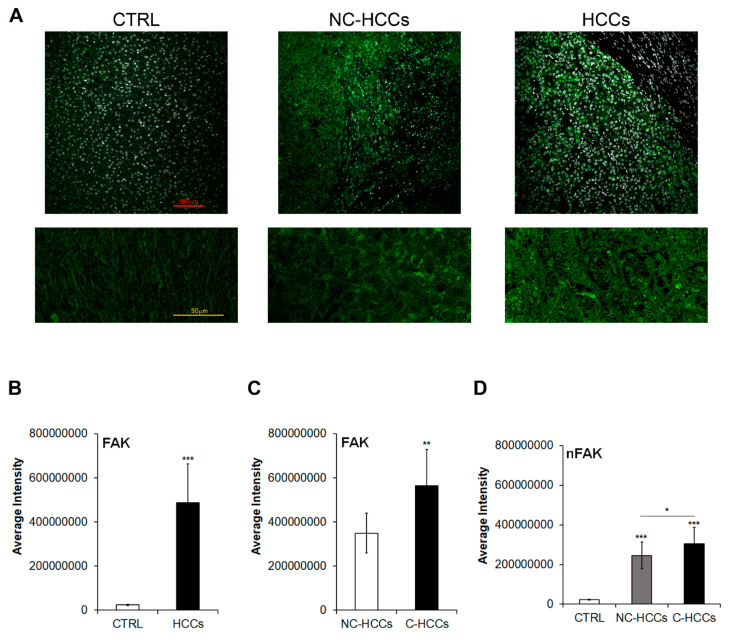
FAK expression and intracellular distribution in healthy livers and pediatric HCCs. (**A**) Representative immunofluorescence of FAK healthy control livers (CTRL), Non-cirrhotic and cirrhotic pediatric HCCs. 20× magnification (upper panels); 60× magnification (lower panels). (**B**) Average fluorescence intensity calculated for FAK in CTRL and HCCs. (**C**) Average fluorescence intensity calculated for FAK in pediatric HCCs according to cirrhotic (C-HCCs)/non-cirrhotic (NC-HCCs) milieu. (**, *p* < 0.01; ***, *p* < 0.001). (**D**) Average fluorescence intensity calculated for nuclear FAK (nFAK) in pediatric HCCs according to cirrhotic (C-HCCs)/non-cirrhotic (NC-HCCs) milieu. (*, *p* < 0.05; **, *p* < 0.01; ***, *p* < 0.001).

We evaluated by immunofluorescence the expression of total FAK in 17 pediatric HCCs (11 HCC in cirrhotic liver, C-HCC, and 6 in non-cirrhotic liver, NC-HCC) compared with 8 age-matched healthy livers (CTRL) (Figure 1A–C and Supplementary Materials Table S1). As shown in Figure 1A,B, the expression of FAK significantly increased (*p* < 0.001) in HCC compared to CTRL. In particular, among HCCs, total FAK was higher in tumors arising in cirrhotic liver (C-HCCs) than in tumors in non-cirrhotic liver (NC-HCCs) (Figure 1C). Interestingly, a similar trend was observed also for the nuclear amount of FAK (Figure 1D).

**Figure 2:**
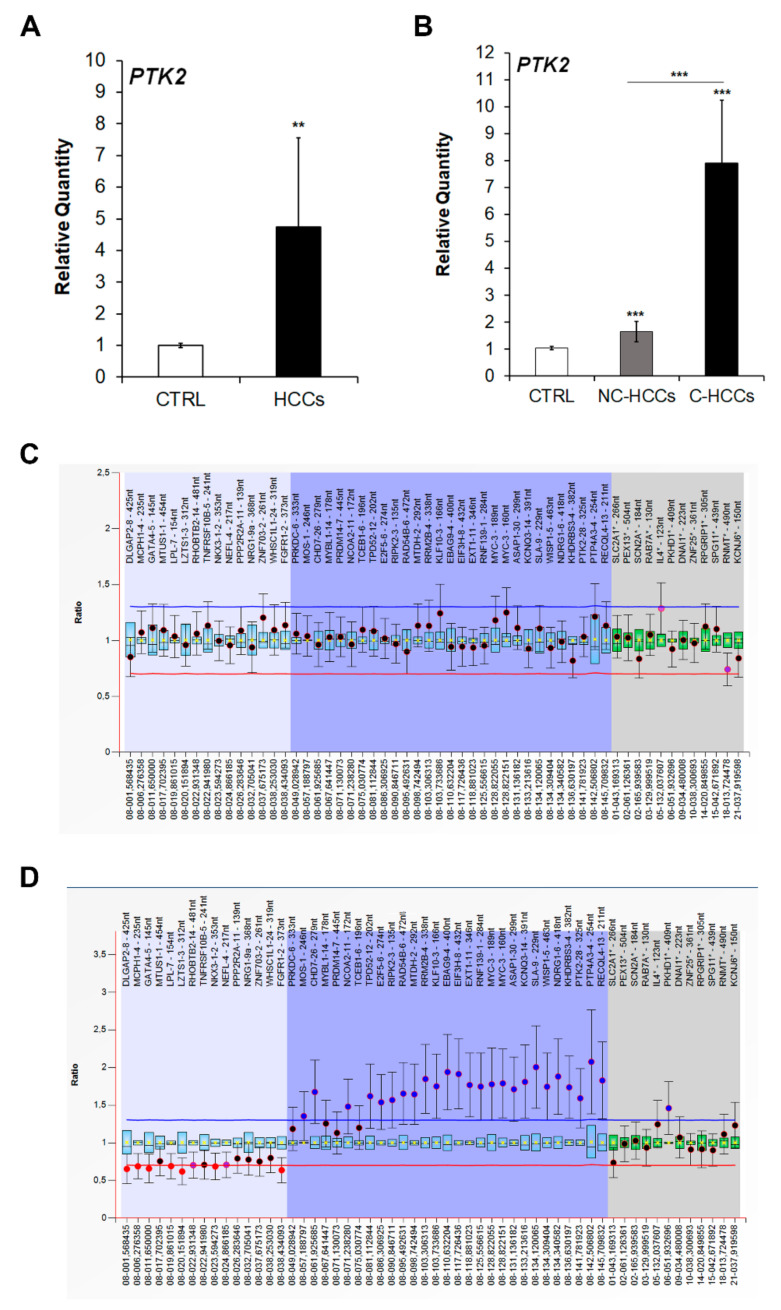
PTK2 over-expression and amplification in pediatric HCCs. Relative mRNA expression of PTK2 gene measured by qRT-PCR in all HCCs vs. CTRL samples (**A**), and in NC-HCCs and C-HCCs vs. CTRL samples (**B**). Normalization was performed with glyceraldehyde-3-phosphate dehydrogenase (GAPDH) mRNA levels. **, *p* < 0.01; ***, *p* < 0.001. MLPA ratio chart of NC-HCC (**C**) and C-HCC (**D**).

Since FAK is encoded by the PTK2 gene, we next quantified the expression of this gene by real-time PCR. As expected, the increased protein levels of FAK in pediatric HCCs was associated to an up-regulation of PTK2 gene expression (Figure 2A). Moreover, PTK2 expression was higher in C-HCCs than in NC-HCCS (Figure 2B). However, by multiplex ligation-dependent probe amplification (MLPA) PTK2 gene duplication was detected only in two C-HCCs (Figure 2C), while it was absent in NC-HCC (Figure 2D) (Supplementary Materials Table S2).

**Figure 3:**
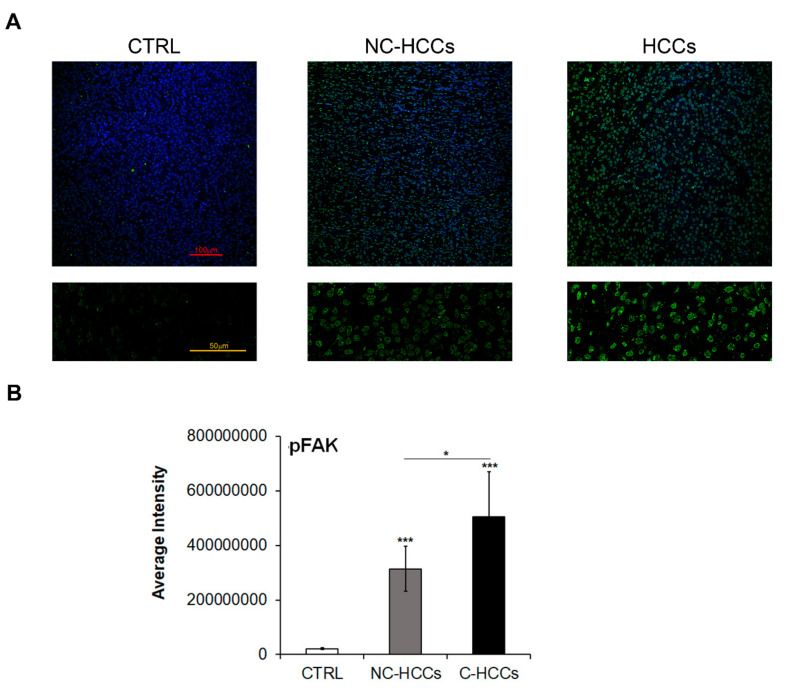
Expression of Y397-pFAK in healthy livers and pediatric HCCs. (**A**) Representative immunofluorescence of FAK healthy livers (CTRL), Non-cirrhotic and cirrhotic pediatric HCCs. 20× magnification (upper panels); 60× magnification (lower panels). (**B**) Average fluorescence intensity calculated for Y397-pFAK in CTRL in pediatric HCCs according to cirrhotic (C-HCCs)/non-cirrhotic (NC-HCCs) milieu. (*, *p* < 0.05; ***, *p* < 0.001).

It is well known that FAK is activated by Y397 autophosphorylation [11]; thus, we also investigated the expression levels of Y397-pFAK (pFAK). As shown in Figure 3A,B, there is a strong up-regulation of pFAK at nuclear levels in all HCCs, and mainly in those from a cirrhotic background.

### 2.2. Total Levels of EZH, H3K27me3 and PCNA Protein Increases in HCCs from a Cirrhotic Background

**Figure 4:**
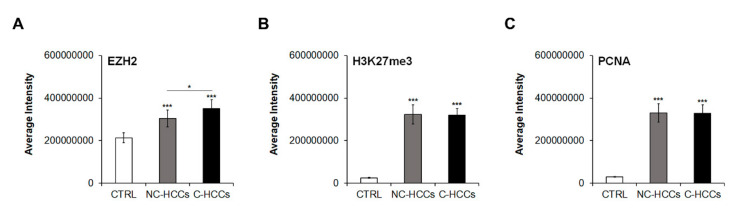
Quantitative imaging of EZH2, H3K27me3, and PCNA in pediatric HCCs. Average fluorescence intensity calculated for (**A**) EZH2, (**B**) H3K27me3, and (**C**) PCNA in CTRL and in pediatric HCCs according to cirrhotic/non-cirrhotic milieu (*, *p* < 0.05; ***, *p* < 0.001 vs. CTRL).

We evaluated by immunofluorescence the expression of total EZH2, H3K27me3, and PCNA in all pediatric HCCs (Supplementary Materials Figure S1). Quantitative analysis of immunofluorescence data (Supplementary Materials Table S1**)** showed that levels of EZH2 were significantly higher in HCC compared to controls with a significant higher expression in C-HCCs than in a non-cirrhotic milieu (Figure 4A). In the same way, H3K27me3 and PCNA were significantly higher in HCC compared to controls, but without relevant differences between HCC in cirrhotic and non-cirrhotic liver (Figure 4B,C).

### 2.3. FAK Expression Correlates with EZH, H3K27me3 and PCNA Protein Increases

**Figure 5:**
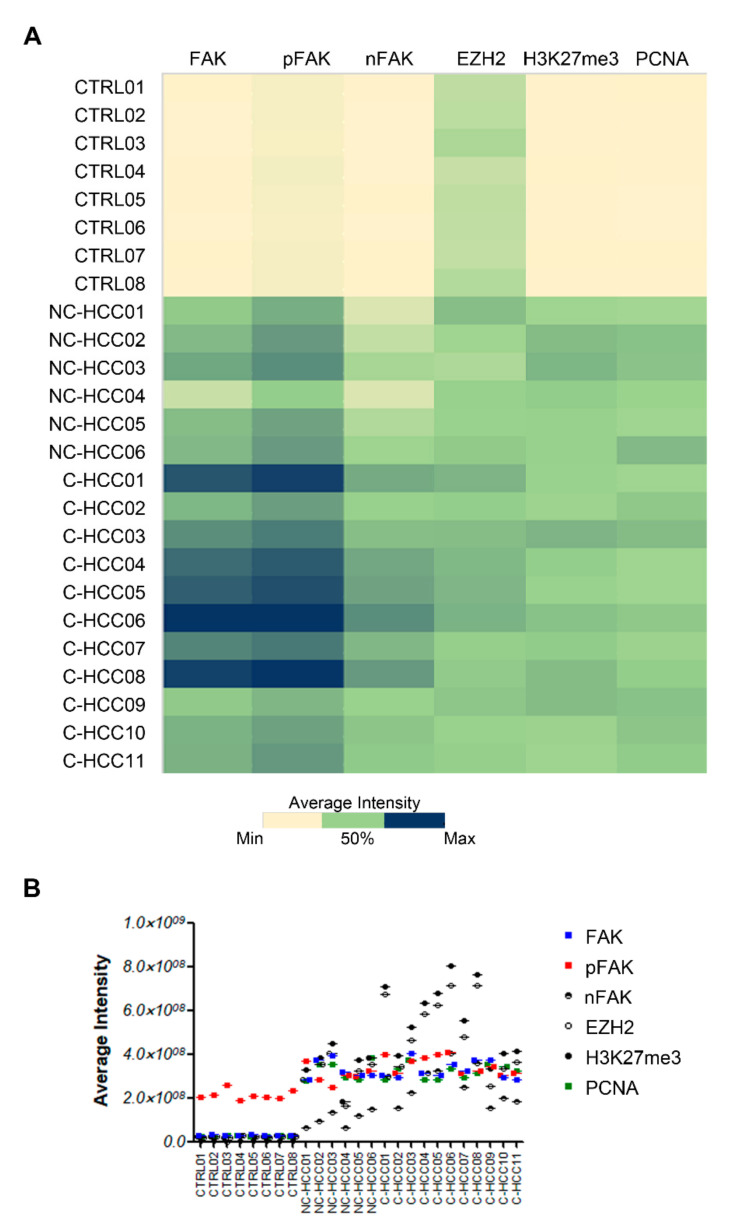
Correlation analysis between FAK, EZH2, H3K27me3, and PCNA in pediatric HCCs. Heatmap representation (**A**) and semiquantitative data (**B**) of the average fluorescence intensity for FAK, pFAK, nFAK, EZH2, H3K27me3, and PCNA in 8 healthy livers and 17 pediatric HCCs.

Figure 5A showed the heatmap representation of average intensity of expression of each protein assayed by immunofluorescence compared with FAK expression in all of samples. Results from Spearman rank correlation analyses among FAK, pFAK, nFAK, EZH2, H3K27me3, and PCNA showed that the expression of each of these markers correlated with each other (Figure 5A and Supplementary Materials Table S3).

### 2.4. The Expression of Total and Nuclear FAK, EZH2, and PCNA Inversely Correlates with Tumor Size

Next, we analysed the correlation between the expression of FAK, EZH2, H3K27me3, and PCNA and clinico-pathological parameters reported in Table 1 and Supplementary Materials Table S4. Spearman’s correlation analysis showed no correlation between the expression of these proteins and metastasis or survival. On the other hand, the expression of total and nuclear FAK, EZH2, and PCNA significantly inversely correlated with tumor size. In fact, Spearman’s correlation coefficient was −0.51 (*p* < 0.05) for total FAK; −0.56 (*p* < 0.05) for nFAK; −0.48 (*p* < 0.05) for EZH2 and −0.51 (*p* < 0.05) for PCNA.

We next performed Spearman’s correlation analysis between the protein expression of total FAK, nFAK, pFAK, EZH2, H3K27me3, and the semi-quantitative scores (score 0 and score 1 to 3) for the expression of nuclear β-Catenin (nβ-Cat) and total GPC3 and GS, which are reported in Supplementary Materials Table S5, Table 2 and Supplementary Materials Figure S2. From this analysis, no significant correlation has emerged.

### 2.5. The Up-Regulation of PTK2 Gene Correlated with that of CTNNB1 Gene

**Figure 6:**
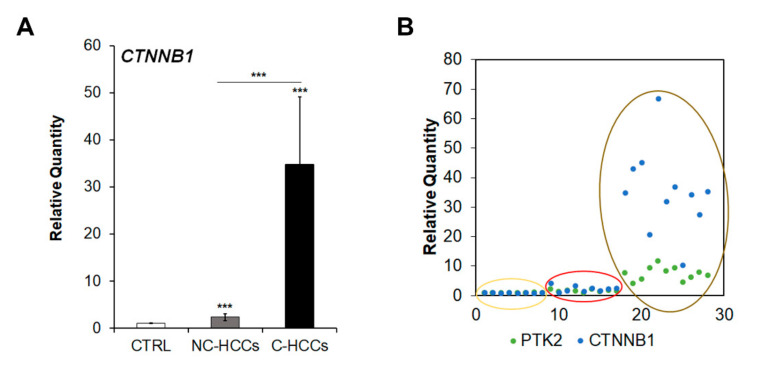
CTNNB1 overexpression in pediatric HCCs. (**A**) Relative mRNA expression of CTNNB1 gene measured by qRT-PCR in NC-HCCs and C-HCCs vs. ***, *p* < 0.01 CTRL samples. (**B**) Relative co-expression of PTK2 and CTNNB1 in CTRL (yellow circle) NC-HCCs (red circle), and C-HCCs (brown circle).

Since Shang et al. [16] have recently reported that concomitant over-expression of PTK2 and CTNNB1 genes resulted in hepatocarcinogenesis in experimental models, we analyzed CTNNB1 gene transcription. As shown in Figure 6A,B, CTNNB1 was up-regulated in cirrhotic (C-HCCs) over-expressing PTK2 compared to non-cirrhotic (NC-HCCs) HCCs.

## 3. Discussion

HCC is a rare tumor with highly aggressive behavior in children. Their morphological and molecular characteristics are quite different in comparison to their adult counterpart [4]. Pediatric HCC may develop in cirrhotic background (as in adult), but frequently onsets in liver without an underlying chronic disease. Our data suggest a possible different cascade of events in carcinogenesis in the two groups.

In our cohort, the results showed an up-regulation of FAK gene/protein expression in pediatric HCCs compared to age-matched pediatric liver tissues. Moreover, FAK overexpression is higher in HCCs from a cirrhotic background than in HCC in non-cirrhotic liver, even if FAK over-expression is not always related to an increased copy number variation of the PTK2 gene. Post-operative recurrences and metastasis are the main reasons for poor prognosis in HCC patients. The adhesion of tumor cells to extracellular matrix (ECM) and vascular endothelial cells, which is mainly mediated through the integrin family of proteins, is a prerequisite for tumor invasion and metastasis. FAK is a downstream signaling molecule for both intra- and extra-cellular signals conveyed through integrins or growth factor receptors. In the cirrhotic liver, the FAK mRNA levels are higher than in the healthy liver [12]. Persistent tissue repair and remodeling due to chronic liver diseases often lead to progressive liver fibrosis without effective treatment. The progression and resolution of liver fibrosis are likely simultaneous processes, involving parenchymal and non-parenchymal cells, activation and apoptosis of hepatic stellate cells (HSCs), inflammation, pro-fibrotic and anti-fibrotic/resolving immune responses, abnormal autophagy, and hepatocyte death and survival [17]. FAK is activated when cells bind to ECM proteins through integrin receptors. An increase of activated FAK seems to play a pivotal role in liver fibrogenesis via activation and differentiation of HSC, promotion of myofibroblastic proliferation and resistance to apoptosis [18]. Interestingly we found that phosphorylated/activated form of FAK is up-regulated at nuclear levels in all HCCs, and mainly in those from a cirrhotic background. Although there is other evidence of nuclear accumulation of FAK in cancers [19], the role of the activated form of this protein into the nucleus of HCC cells remains to be explored.

In a previous work, we demonstrated that FAK silencing reduced in vitro and in vivo HCC growth [10]. In the same study we also found that PCNA, EZH2, and H3K27me3 were concomitantly up-regulated in the analysis of tissue arrays of human adult HCCs. Accordingly, in the current study the expression of these three proteins was up-regulated in HCCs with respect to healthy livers, but only EZH2 was significantly increased in cirrhotic compared to non-cirrhotic HCCs. These results are in agreement with several previous studies that revealed dysregulation of EZH2 in human HCC [20,21]. In particular, it has been reported that this epigenetic regulator has pro-oncogenic and pro-metastatic roles in HCC [20]. EZH2 gene/protein is frequently upregulated in primary HCC and this correlates to poor prognosis of HCC: Metastatic HCC features, including the presence of venous invasion, direct liver invasion, and the absence of tumor encapsulation [21,22]. Furthermore, Gao et al. [7] demonstrated that EZH2 may inhibit the expression of target genes in HCC through H3K27-dependent and H3K27-independent mechanisms. Moreover, increased H3K27me3 in HCC significantly correlated with large tumor size, poor differentiation, advanced clinical stage, vascular invasion as well as shorter survival of HCC patients. Therefore, the dysregulated EZH2-H3K27me3 epigenetic mark has a profound role in hepatocarcinogenesis [8]. In agreement with these findings, our results suggest that aberrant over-expression of EZH2 and H3K27me3 could be involved in tumor aggressiveness also in pediatric HCCs.

In adults, HCC with infiltrative growth was more frequent in patients with higher than in those with lower FAK expression [10]. In pediatric HCC, we did not find a significant correlation between FAK expression and other features of aggressiveness, such as tumor multifocality, histologic type of tumor, capsular infiltration, vascular invasion, or metastasis. However, this apparent discrepancy with adult HCC is probably due to the small number of cases and to the presence of only two HCCs of infiltrative type (#2 and #5) in our series, while other HCCs were of the nodular type. Interestingly, in our cohort, the expression of FAK, EZH2, H3K27me3, and PCNA inversely correlated with tumor size: Higher expression of these proteins was found in smaller cirrhotic HCCs. Intriguingly, in our cohort, there was an apparently inverse correlation between tumor size and FAK, EZH2, H3K27me3, and PCNA expression. In our opinion, this is mostly related to the dichotomy between NC-HCCs and C-HCCs and with higher expression mostly found in the cirrhotic group, which was also characterized by an overall small size of the lesion, probably related to the close follow-up observed for these patients because of their pathology and an early detection of the mass. Besides, it is possible that FAK does not influence the development and diffusion of HCC in non-cirrhotic livers, which are much more represented in the pediatric population compared to adults.

Activation of FAK can target multiple downstream signaling pathways that regulate cell proliferation, survival, and cell migration. Shang et al. [23] demonstrated that FAK deletion/inhibition may block tumor progression and delays mortality of mice with a mesenchymal–epithelial transition factor (c-MET)/β-catenin-driven HCC. These findings suggest that combination of the c-MET and β-catenin oncoproteins may exert a synergistic effect on FAK activation. Indeed, more recently, the same authors reported that co-expression of FAK and β-catenin was able to induce HCC in mice [16]. We found that nuclear expression of β-Catenin was prevalent in NC-HCCs (4/6 cases), although apparently not related to a mutation of CTNNB1, in contrast with the assumption of a correlation between nuclear positive immunostaining for β-Catenin and mutations of CTNNB1 gene. However, accordingly with data reported in experimental models [16], we found an over-expression of CTNNB1 and PTK2 in pediatric HCCs and mainly in those from cirrhotic livers, confirming the hypothesis of a cooperation of these genes during hepatocarcingenesis.

## 4. Materials and Methods

### 4.1. Tumor Specimens and Histology

A retrospective review of patients with HCC was conducted after approval from the institutional review board (protocol number OPBG_768.12). From 2009 to 2018, 17 HCC were identified from the pathology database: 12 explanted livers, 3 right hepatectomy, and 2 liver biopsies. The clinico-pathological features of the pediatric HCCs included in this study are reported in Supplementary Materials Table S2. Fourteen patients were alive, 2 died for the natural progression of untreatable/non-responder HCC, and 1 died after liver transplantation. Eight control livers were obtained from donors (4 male and 4 female, age range from 2 months to 17 years) at the time of transplantation.

Formalin-fixed and paraffin embedded (FFPE) tissue 3 µm-thick sections were stained with hematoxylin and eosin (H and E), period acid-Schiff (PAS), period acid-Schiff after diastase digestion (PASD), and Masson trichrome using routine methods in liver pathology. All slides were reviewed by two pathologists (PF and RA).

### 4.2. Immunohistochemistry

The immunohistochemical panel includes the antibodies currently used for the diagnostic work up of HCC: anti-β-catenin (monoclonal Ab, 1:100 working dilution, clone 17C2, Leica Biosystems, Germany), anti-Glypican3 (monoclonal Ab, 1:1000 working dilution, clone 1G12, BioMosaics Inc., Burlington, VT), and anti-Glutamine Synthetase (monoclonal Ab, 1:400 working dilution, clone GS6, Millipore Inc., Billerica, MA, USA). The immunoreactivity for β-Cat (nuclear staining), GPC3 and GS (cytoplasmic staining) was semiquantitatively analyzed for percentage of positive cells by a visual score calculated in at least 10 representative fields). Score 0 (<5%); 1 (16–30%); 2 (31–60%); and 3 (>60%).

### 4.3. Immunofluorescence

The liver tissue was fixed in 10% buffered formalin. Immunostaining of formalin-fixed paraffin-embedded tissue specimens was performed on 2 μ sections. After dewaxing and rehydrating, heat-induced epitope retrieval was performed by boiling the slides with Dako Target Retrieval Solution EDTA (pH 9) (Dako, Glostrup, Denmark). The primary antibodies were added and incubated overnight at +4 °C (1:200 anti-FAK rabbit monoclonal antibody (US Biological Life Science, Swampscott, MA, USA); 1:300 anti-H3K27me3 rabbit monoclonal antibody (Cell Signaling Technology, Danvers, MA, USA); 1:100 anti-EZH2 mouse monoclonal antibody (BD Biosciences, USA); 1:300 anti-PCNA rabbit monoclonal antibody (Santa Cruz Biotechnology, Dallas, TX, USA). The primary antibody was revealed with the secondary antibody Alexa Fluor (Applied Biosystems, Life Technologies, Carlsbad, CA, USA). The confocal microscopy imaging was performed on Olympus Fluoview FV1000 confocal microscope equipped with FV10-ASW version 2.0 software, using 20× and 40× objective. Optical single sections were acquired with a scanning mode format of 1024 × 1024 pixels, sampling speed of 40 μs/pixel, and 12 bits/pixel images. Laser’s power, beam splitters, filter settings, pinhole diameters, and scan mode were the same for all examined samples of each staining. Fluorochromes unmixing was performed by acquisition of automated-sequential collection of multi-channel images, in order to reduce spectral crosstalk between channels.

For quantification of expression were used at least eight free area from single section that was used to manually draw the region of interest (ROI). The intensity average of fluorescence was calculated using ImageJ software and the values of average intensity for each sample were graphed with heatmap corresponding to two-dimensional matrices in which cells colors depend on the cell value by using Microsoft Excel 2016.

### 4.4. Real-Time Quantitative Reverse Transcription PCR

Total RNA extraction was performed using ReliaPrep FFPE Total RNA miniprep system (Promega, Milan, Italya) according to the manufacturer’s protocol. cDNA was synthesized by The SuperScript VILO cDNA Synthesis Kit (Thermo Scientific, Waltham, MA, USA) according to kit’s instructions. Quantitative Real-Time (qRT-PCR) amplification, detection and analysis was performed by ABI Prism 7900HT Fast Real-Time PCR System (Applied Biosystems, Foster City, CA, USA) using SensiFast PCR Master Mix (2X) No AmpErase^®^ UNG (Applied Biosystems, Foster City, CA, USA). The samples were normalized according to the GAPDH. TaqMan gene assay for GAPDH and CTNNB1, were purchased from Applied Biosystems, (Foster City, CA, USA); The TaqMan probes for human FAK was supplied by IDT (Integrated DNA Technologies, Coralville, IA, USA). Based on the ΔΔCt method, relative amounts of mRNA were expressed as fold changes versus control.

### 4.5. Multiplex Ligation-Dependent Probe Amplification (MLPA) Analysis

*PTK2* (FAK) was investigated using MLPA technique [24]. The P014 MLPA kit (MRC Holland, Amsterdam, The Netherlands) was employed. This kit includes 44 probes. MLPA analyses were performed in accordance with the manufacturer’s instructions. The results were analyzed using Coffalyser.net software (MRC Holland, Amsterdam, The Netherlands).

### 4.6. Mutation Screening

Genomic DNA from FFPE HCC and paired normal liver tissue, extracted using NucleoSpin™ Tissue (Macherey-Nagel, Düren, Germany), was available in 10/17 cases (Supplementary Materials Table S5). Mutation screening was done using polymerase chain reaction (PCR) amplification and DNA sequencing of exons 3, 7, and 8 and relative splice junctions of CTNNB1 (NM_001098209) (primer sequence upon request).

### 4.7. Statistical Analysis

All data are presented as mean ± standard error of the mean. Spearman correlation test was performed to test the association between protein expression and histopathological features on two-way contingency tables. Statistical significance was calculated using the Student’s t-test. *p* < 0.05 was considered to be significant. Statistical analysis was undertaken using GraphPad Prism 5 (GraphPad Software Inc., San Diego, CA, USA).

## 5. Conclusions

We showed that pediatric HCC may develop either in a background of cirrhotic disease or as a de novo tumor in healthy liver. The molecular profile appears different in the two conditions. FAK, EZH2 and H3K27me3 exhibited a significantly higher expression in cirrhotic compared to non-cirrhotic HCCs. Further studies are needed to explore their prognostic significance in pediatric population. However, the association with cirrhosis supports a different pathogenetic mechanism respect to sporadic HCCs

## Figures and Tables

**Table 1:** Clinico-Pathological Indicators and Survival Conditions of Pediatric HCCs.

	NC-HCCs (*n* = 6)	C-HCCs (*n* = 11)
Age, mean ± S.D (years)	8.7 ± 4.4	6.4 ± 4.0
Sex, F/M	1/5	6/5
Tumor size, mean ± S.D (cm)	7.0 ± 2.7	5.5 ± 3.5
Metastasis (%)	33.3	-
Survival, death (%)	16.6	18.2

**Table 2:** nβ-Cat, GPC3, and GS Protein Expression (Score 0–3) in all 17 Patients Tested. Score 0, ≤5% Positive Tumor Cells; Score 1, 6–30% Positive Tumor Cells; Score 2, 31–60% Positive Tumor Cells; Score 3, >60% Positive Tumor Cells.

Score	nβ-Cat	GPC3	GS
0	13	7	1
1	2	5	3
2	1	3	3
3	1	2	10

## Supplementary Materials

The following are available online at https://www.mdpi.com/1422-0067/21/16/5795/s1.
