# Supplementary material for: Focal Adhesion Kinase (FAK) Over-Expression and Prognostic Implication in Pediatric Hepatocellular Carcinoma
Source: Int J Mol Sci. 2020 Aug 12;21(16):5795. doi: 10.3390/ijms21165795 (PMC7460809; doi:10.3390/ijms21165795)

Table S1. Average intensity data obtained by imaging analysis.

Sample	FAK	pFAK	nFAK	EZH2	H3K27me3	PCNA
CTRL01	25410256	20025645	1778717	205636958	25456965	28565856
CTRL02	23124585	21548563	1387475	215685968	23654585	31256369
CTRL03	20145236	18457896	604357	258696547	28450325	30256585
CTRL04	28414236	26145632	1136569	186596586	27451263	29565485
CTRL05	24102563	22154763	2892307	210363526	22145263	33256585
CTRL06	21365485	16554103	1068274	201452639	21056365	27523654
CTRL07	26456369	24789632	2645636	195847215	26365690	29526545
CTRL08	27458630	23568412	2745863	235263658	24521457	30256365
NC-HCC01	327053800	285645897	65410760	366365785	276052456	285265155
NC-HCC02	382809076	355654253	95702269	284574536	354120365	374589633
NC-HCC03	447081442	401562496	134124432	250653636	352541563	394524556
NC-HCC04	183706416	165620145	64297245	303696985	293562545	320545632
NC-HCC05	372203056	324514865	119104977	296585663	284136523	304512563
NC-HCC06	385645852	352015632	146545423	325636586	382545658	302565635
C-HCC01	706632560	675456587	296785675.2	398748596	284596325	301457855
C-HCC02	391025653	344565810	152500004.7	312565847	335415258	295654788
C-HCC03	522042485	463012530	224478268	370265888	374563254	401547853
C-HCC04	635458632	584015230	311374729	384574896	285463651	314521455
C-HCC05	676433921	625463564	324688282	397896545	284575963	301452654
C-HCC06	805264780	714236520	402632390	405936856	332565452	354155236
C-HCC07	554585630	476541254	249563533	312563674	295451563	321025455
C-HCC08	765489990	713015826	359780295	325614755	314587896	374578452
C-HCC09	331134418	254869457	152321832	342563651	354156365	374512447
C-HCC10	405663254	331042519	198774994	302563685	345265558	295451632
C-HCC11	412563521	364865987	181527949	312563658	321245874	285456521

Table S2. MLPA analysis on pediatric HCCs.

	8p		8q	
			PTK2	
**NC-HCC01**	1	1	1	1
**NC-HCC02**	1	1	1	1
**NC-HCC03**	1	1	1	1
**NC-HCC04**	1	1	1	1
**NC-HCC05**	1	1	1	1
**NC-HCC06**	-	-	-	-
**C-HCC01**	-	-	-	-
**C-HCC02**	0.5	2	1.5	2.5
**C-HCC03**	0.5	1.5	1.5	2
**C-HCC04**	1	1	1	1
**C-HCC05**	1	1	1	1
**C-HCC06**	1	1	1	1
**C-HCC07**	0.5	1.5	1	1.5
**C-HCC08**	1	1	1	1
**C-HCC09**	1	1	1	1
**C-HCC10**	1	1	1	1
**C-HCC11**	-	-	-	-

Table S3. Correlations of the protein expression: Spearman rank correlation tests.

		FAK	pFAK	nFAK	EZH2	H3K27me3	PCNA
FAK	*Correlation Coefficient*	1	0.987	0.962	0.786	0.708	0.634
	P value	<0.0001	<0.0001	<0.0001	<0.0001	<0.0001	0.0006
pFAK	*Correlation Coefficient*	0.987	1	0.940	0.787	0.741	0.636
	P value	<0.0001		<0.0001	<0.0001	<0.0001	0.0006
nFAK	*Correlation Coefficient*	0.962	0.940	1	0.843	0.687	0.617
	P value	<0.0001	<0.0001		<0.0001	0.0001	0.0010
EZH2	*Correlation Coefficient*	0.786	0.787	0.843	1	0.619	0.578
	P value	<0.0001	<0.0001	<0.0001		0.0009	0.0024
H3K27me3	*Correlation Coefficient*	0.708	0.741	0.687	0.619	1	0.803
	P value	<0.0001	<0.0001	0.0001	0.0009		<0.0001
PCNA	*Correlation Coefficient*	0.634	0.636	0.617	0.578	0.803	1
	P value	0.0006	0.0006	0.0010	0.0024	<0.0001	

Table S4. Available clinics of samples.

Sample	Medical History	Age	Sex	Tumor size (radiologic)	Metastasis	Therapy	Surgery	Outcome
NC-HCC01	No Cirrhosis	8y	M	multinodular, max 3 cm	NEG	PLADO + Bevacizumab	LT	alive
NC-HCC02	No Cirrhosis	10y	M	9 cm	NEG	PLADO + Bevacizumab	LT	alive
NC-HCC03	No Cirrhosis	16y	F	multinodular, max 3.5 cm	POS (lung)	PLADO + Bevacizumab	-	dead
NC-HCC04	No Cirrhosis	6y	M	multinodular, max 7 cm	NEG	PLADO	RH	alive
NC-HCC05	No Cirrhosis	9y	M	10 cm	NEG	PLADO + Bevacizumab	LT	alive
NC-HCC06	No Cirrhosis	3y	M	6 cm	POS	PLADO + Bevacizumab	RH	alive
C-HCC01	PFIC2	2y	F	1.5 cm	NEG	-	LT	NA
C-HCC02	Tyrosinemia	8y	M	1.5 cm	NEG	-	LT	alive
C-HCC03	Tyrosinemia	10y	F	3 cm	NEG	-	LT	alive
C-HCC04	Tyrosinemia	11y	M	1.2 cm	NEG	-	LT	alive
C-HCC05	Tyrosinemia	5y	M	2.4 cm	NEG	-	LT	alive
C-HCC06	Metabolic Cirrhosis	9y	F	1.4 cm	NEG	-	LT	alive
C-HCC07	HBV	10y	M	1.5 cm	NEG	-	LT	alive
C-HCC08	Cardiac Cirrhosis	25y	F	8 cm	NEG	-	-	dead
C-HCC09	Cardiac Cirrhosis	33y	F	4.5 cm	NEG	-	RH	alive
C-HCC10	Neonatal Hemochromatosis	1mo	F	2.3 cm	NEG	-	LT	dead
C-HCC11	Biliary Atresia	10mo	M	1.3 cm	NEG	-	LT	alive

HBV, hepatitis B virus; LT, liver transplantation; mo, months; PLADO, cisplatin/doxorubicin; RH, Right hepatectomy; y, years

Table S5. Data on histochemical scores for the immunohistochemical staining in pediatric HCCs.

Sample	nβ-Cat	GPC3	GS	β-Cat Mutation
CTRL01	-	-	-*	NA
CTRL02	-	-	-*	NA
CTRL03	-	-	-*	NA
CTRL04	-	-	-*	NA
CTRL05	-	-	-*	NA
CTRL06	-	-	-*	NA
CTRL07	-	-	-*	NA
CTRL08	-	-	-*	NA
NC-HCC01	-	++	++	NA
NC-HCC02	++	-	+++	-
NC-HCC03	+	-	+++	-
NC-HCC04	+	-	-	-
NC-HCC05	-	+	+++	-
NC-HCC06	+++	+++	+++	NA
C-HCC01	-	+	++	NA
C-HCC02	-	+++	+++	-
C-HCC03	-	++	+++	-
C-HCC04	-	-	+++	-
C-HCC05	-	+	+++	-
C-HCC06	-	-	++	-
C-HCC07	-	+/-	+/-	NA
C-HCC08	-	++	+	NA
C-HCC09	-	-	+	-
C-HCC10	-	+	+++	NA
C-HCC11	-	+/-	+++	NA

*Only perivascular positivity.

Supplementary Figure 1. Representative imaging of immunofluorescence for EZH2, H3K27me3 and PCNA.

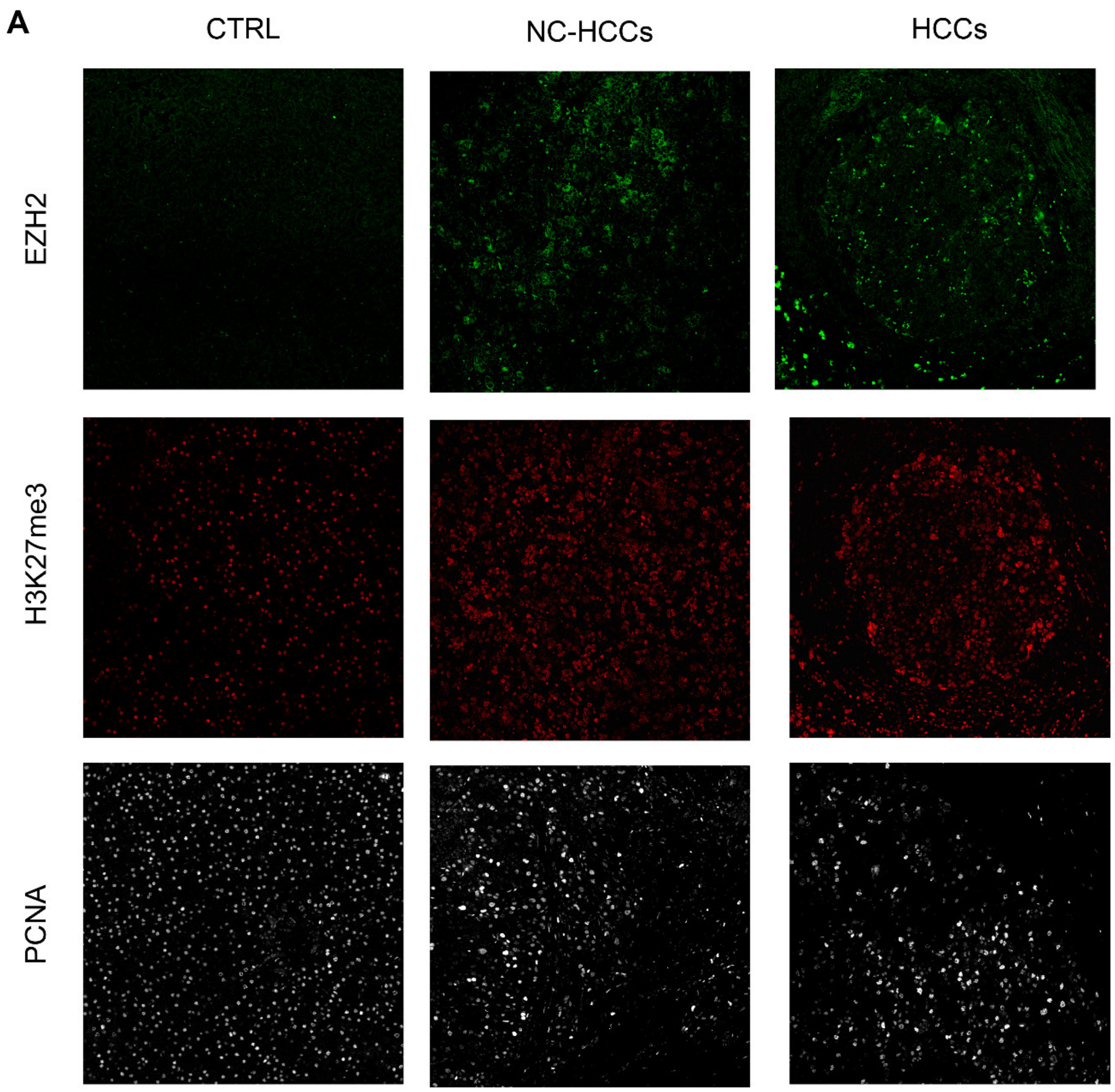

Supplementary Figure 2. Representative imaging of immunohistochemical staining for hematoxylin and eosin (H&E), β-Catenin and GPC3 and GS.

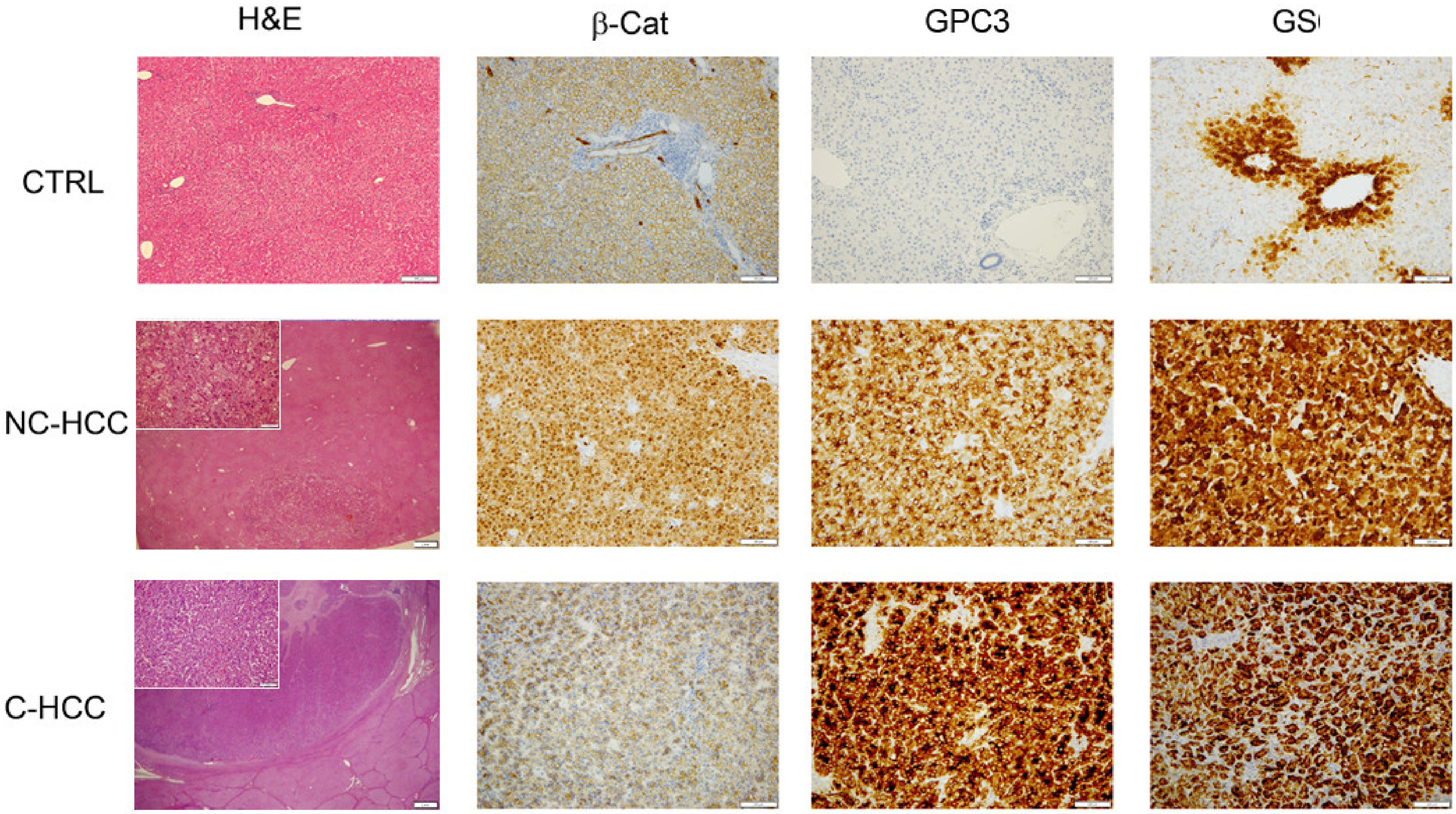

Supplement: Supplementary file 1 [file ijms-21-05795-s001.pdf]
